# Protective role of all-trans retinoic acid (ATRA) against hypoxia-induced malignant potential of non-invasive breast tumor derived cells

**DOI:** 10.1186/s12885-018-5038-6

**Published:** 2018-11-29

**Authors:** Yasamin Al-Qassab, Silvia Grassilli, Federica Brugnoli, Federica Vezzali, Silvano Capitani, Valeria Bertagnolo

**Affiliations:** 1 Signal Transduction Unit, Section of Anatomy and Histology, Department of Morphology, Surgery and Experimental Medicine, University of Ferrara, Via Fossato di Mortara, 70, 44121 Ferrara, Italy; 2 College of Medicine, Department of Anatomy, University of Baghdad, Baghdad, Iraq; 3 LTTA Centre, University of Ferrara, Ferrara, Italy

**Keywords:** DCIS, Hypoxia, Tumor progression, ATRA, PLC-β2

## Abstract

**Background:**

The presence of hypoxic areas is common in all breast lesions but no data clearly correlate low oxygenation with the acquisition of malignant features by non-invasive cells, particularly by cells from ductal carcinoma in situ (DCIS), the most frequently diagnosed tumor in women.

**Methods:**

By using a DCIS-derived cell line, we evaluated the effects of low oxygen availability on malignant features of non-invasive breast tumor cells and the possible role of all-trans retinoic acid (ATRA), a well-known anti-leukemic drug, in counteracting the effects of hypoxia. The involvement of the β2 isoform of PI-PLC (PLC-β2), an ATRA target in myeloid leukemia cells, was also investigated by specific modulation of the protein expression.

**Results:**

We demonstrated that moderate hypoxia is sufficient to induce, in DCIS-derived cells, motility, epithelial-to-mesenchymal transition (EMT) and expression of the stem cell marker CD133, indicative of their increased malignant potential.

Administration of ATRA supports the epithelial-like phenotype of DCIS-derived cells cultured under hypoxia and keeps down the number of CD133 positive cells, abrogating almost completely the effects of poor oxygenation. We also found that the mechanisms triggered by ATRA in non-invasive breast tumor cells cultured under hypoxia is in part mediated by PLC-β2, responsible to counteract the effects of low oxygen availability on CD133 levels.

**Conclusions:**

Overall, we assigned to hypoxia a role in increasing the malignant potential of DCIS-derived cells and we identified in ATRA, currently used in treatment of acute promyelocytic leukemia (APL), an agonist potentially useful in preventing malignant progression of non-invasive breast lesions showing hypoxic areas.

## Background

Hypoxia is defined as a reduced oxygen availability and solid tumors, including breast cancer, often contain hypoxic regions because of their rapid and uncontrollable cell proliferation combined with a structurally and functionally abnormal vasculature [1]. Low oxygen availability generates a hostile microenvironment in which tumor cells activate adjustment mechanisms in order to survive. Adaptation of neoplastic cells is a crucial driving force in the progression towards a more aggressive and resistant tumor phenotype [2–5]. Key mediators in this phenomenon are the hypoxia-inducible factors (HIFs), able to modulate the expression of genes involved in almost all key step of tumor progression as survival, angiogenesis, metabolic reprogramming, immortalization, EMT, stem cell maintenance, resistance to radiation and chemotherapy, invasion and metastasis [6].

Even if the presence of hypoxic areas is common in all breast lesions, no data clearly correlate low oxygenation with the acquisition of malignant features by non-invasive cells, particularly by cells from ductal carcinoma in situ (DCIS), that constitutes the 20–25% of newly diagnosed breast cancers in industrialized countries [7, 8].

In recent years, retinoids, active metabolites of vitamin A, have been proven promising agents in the management of solid tumors, exerting their function on cell growth and regulating mitochondrial permeability, death receptors, ubiquitination, and oxygen reactive species [9, 10]. The biological activity of retinoids is primarily mediated by activation of nuclear retinoid-receptors, ligand-activated transcription factors, which are grouped into RARs and RXRs families [9]. All-*trans* retinoic acid (ATRA), a well-known anti-leukemic drug [11, 12], is the only example of a clinically useful cyto-differentiating agent in treatment of some solid tumors, resulting less toxic and more specific than conventional chemotherapy [13, 14]. In cells from invasive breast tumors ATRA acts preferentially by decreasing proliferation and increasing differentiation and apoptosis, mainly through its nuclear RARα [15, 16]. Moreover, the pleotropic effects of ATRA in breast cancer cells were also correlated to non-genomic and multi-layered pathways also aimed to target the cancer stem cells-like population [17, 18].

Among the molecules up-modulated by ATRA in leukemic cells, the beta 2 isoform of the phosphoinositide-dependent phospholipase C (PLC-β2) is ectopically expressed in primary invasive breast tumors in which it strongly correlates with malignancy and poor prognosis [19]. PLC-β2 is also expressed in invasive breast tumor-derived cell lines with different phenotypes, in which it sustains invasion capability [20]. In low invasive breast tumor derived cells, PLC-β2 is down-modulated by low oxygen availability and its over-expression prevents the hypoxia-induced increase of cells showing high surface levels of the cancer stem cell marker CD133 [21].

Aim of this study was to assess if low oxygen availability induces malignant properties in cells derived from DCIS and to establish whether ATRA, possibly through up-modulation of PLC-β2, may counteract the impact of hypoxia in non-invasive breast cancer cells.

## Methods

All reagents were from Sigma (St Louis, MO) unless otherwise indicated.

### Cell culture and reagents

The breast cancer-derived cell line MCF10DCIS, kindly provided and characterized by Dr. Macpherson (Beatson Institute for Cancer Research, Glasgow, UK), was cultured in Advanced DMEM/F12 medium (Gibco Laboratories, Grand Island, NY), 1% L-Glutamine, 5% horse serum (HS, Gibco Laboratories) and 1% penicillin-streptomycin solution (Gibco Laboratories) and grown at 37 °C in a humidified atmosphere of 5% CO_2_ in air. Sub-confluent cells were counted daily, maintained between 2 × 10^5^/cm^2^ and 3 × 10^5^/cm^2^ and cell morphology was evaluated using an inverted phase-contrast microscope (Nikon, Melville, NY).

Exposure of cell cultures to hypoxia (1% O_2_) was performed in Forma™ Series II Water Jacketed CO_2_ Incubator (Thermo Fisher Scientific Inc., Waltham, MA).

Increasing concentrations of ATRA (0.1 μM, 1 μM, 10 μM) dissolved in DMSO were administered to MCF10DCIS cells grown at both normoxia and hypoxia for 4 days.

Cells in all experimental conditions were daily counted by means of a hemocytometer in the presence of trypan blue, in order to determine the number of viable cells.

The morphology of cells under the different experimental conditions was analyzed with an inverted phase-contrast microscope (Nikon Eclipse TE2000-E, Nikon S.p.a., Florence, I). Cell images were acquired using the ACT-1 software for the DXM1200F digital camera (Nikon) and analyzed with the ImageJ software (http://rsb.info.nih.gov/ij/), as previously reported [22]. For each experimental condition, 3 different areas containing at least 100 cells were analyzed and cells were defined “elongated” when their longest axis was at least 2 times larger than their shortest axis.

### Immunochemical and immunocytochemical analysis

Total cell lysates were separated on 7.5% polyacrylamide denaturating gels and blotted to nitrocellulose membranes (GE Healthcare Life Science, Little Chalfont, UK). The membranes were reacted with antibodies directed against HIF-1α, CAIX, RARα, Vimentin, E-Cadherina and SLUG (Santa Cruz Biotechnology) and β-tubulin (Sigma), as previously reported [21]. The immunocomplexes were detected by chemiluminescence using the ECL system (Perkin-Elmer, Boston, MA), according to the manufacturer’s instructions. The chemiluminescence derived bands were acquired with an ImageQuantTM LAS 4000 biomolecular imager (GE Healthcare Life Science) and the densitometrical analysis was performed by means of Image Quant TL software (GE Healthcare Life Science).

Immunocytochemical analysis of PLC-β2, HIF-1α and β-catenin was performed essentially as previously described [21]. In particular, cells grown onto glass slides under different experimental conditions were fixed with freshly prepared 4% paraformaldehyde, washed once in PBS, incubated with the primary antibodies (Santa Cruz Biotechnology) for 3 h in NET gel at room temperature and then reacted with a FITC-conjugated secondary antibody diluted in NET gel. Fluorescent samples were analyzed with a Nikon Eclipse TE2000-E microscope (Nikon), acquiring cell images by the ACT-1 software for a DXM1200F digital camera (Nikon). To measure flurescence staining, digitized images were analyzed with the ImageJ software, following the manufacturer’s instructions (http://rsb.info.nih.gov/ij/).

### Real-time assays of cell migration and invasion

Cells were subjected to migration and invasion assays under normoxic and hypoxic culture conditions by means of the xCELLigence RTCA system (Real-Time Cell Analyzer System, Acea Biosciences Inc., San Diego, CA), developed to monitor cell events in real time, without incorporation of dyes, by measuring electrical impedance, essentially as previously reported [21]. For migration assays, 4 × 10^5^ cells ∕ well were seeded onto the top chambers of CIM-16 plates and the bottom chambers were filled with medium containing 5% FBS as chemoattractant. For analysis of invasiveness, the upper side of the upper chambers were covered with a layer of diluted Matrigel (BD Biosciences, 1:20 and 1:40) and the bottom chambers were filled with medium containing 10% FBS. For both migration and invasion assays, each condition was performed in quadruplicate and the signal detection was programmed every 15 min for a total of 24 h. Impedance values were expressed as a dimensionless parameter termed Cell Index (CI). The rate of cell migration and invasion was also determined by calculating the slope, that describes the steepness, incline, gradient, and changing rate of the Cell Index curves over time.

To measure migration and invasion under hypoxia, all the procedures described above were performed by allocating the RTCA station inside the incubator, in which the oxygen concentration was 1% for the entire duration of the experiment.

### Cytofluorimetrical evaluation of CD133 expression

CD133 surface expression was evaluated by flow cytometry by direct staining of cells with a phycoerythrin (PE)-conjugated anti-CD133/2 monoclonal antibody (293C3, Miltenyi Biotec, Bologna, I), following a previously reported procedure [21, 23]. In particular, 5 × 10^5^ cells, under the different experimental conditions, were incubated with 10 μl of CD133-PE antibody in 100 μl of PBS for 10 min at 4 °C in the dark. After washing with cold PBS, a solution containing 7-amino-actinomycin D (BD Biosciences, San Josè, CA) was added to the samples to identify dead cells to be excluded from the analysis, following manufacturer’s protocol. Non-specific fluorescence was assessed by using an isotype-matched control, Mouse IgG1-PE (Immunotech, Coulter Company, Marseille, F). All samples were analyzed by a FACS Calibur flow cytometer (BD Biosciences) with CellQuest Pro 6.0 software (BD Biosciences). Data collected from 10,000 cells are shown as a percentage of positive cells or as Mean Fluorescence Intensity (MFI) values.

### Quantitative analysis of PLC-β2 mRNA and down-modulation of PLC-β2 expression

High-quality total RNA from cells under different experimental conditions was extracted with RNeasy® Micro Kit (Qiagen S.P.A., Milan, I), as previously reported [21]. The first-strand cDNA was synthesized from RNA using the ImProm-II™ Reverse Transcription System kit (Promega, Madison, WI). The cDNA synthesis was carried out by using the GeneAmp® PCR System 2700 thermal cycler (Thermo Fisher Scientific). The obtained cDNAs were employed as template for quantitative Real-Time PCR for PLC-β2 measurement using the TaqMan® Gene Expression Assay (Thermo Fisher Scientific). Thermal cycling and fluorescence detection were performed according to the manufacturer′s instruction, using a Bio-Rad CFX96™ sequence detection system (Bio-Rad Laboratories, Hercules, CA) and the data were analyzed by using a dedicated software (Bio-Rad). Quantification of mRNA expression was calculated by the 2^-∆CT^ method and normalized to the expression of RPL13A mRNA levels.

The down-modulation of PLC-β2 expression was performed by silencing the protein with a pool of 3 target-specific 20-25 nt siRNAs designed by Santa Cruz Biotechnology (Santa Cruz) and transfection was conducted with 1 mg/ml Lipofectamine 2000 (Invitrogen), as previously reported [21]. As a control of transfection efficiency, a non-silencing fluorescein-labeled duplex siRNA, purchased from Qiagen, was used. Transfected cells were incubated at 37 °C in a 5% CO_2_ atmosphere in growing medium under normoxic and hypoxic conditions prior to RNA extraction and cellular assays.

### Statistical analysis

Statistical analysis was performed by using the non-parametric Mann-Whitney U test for independent samples (SPSS Inc., Chicago, IL). Two-sided tests were used and *P*-values < 0.05 were considered statistically significant.

## Results

### Hypoxia induces malignant properties in DCIS-derived cells

**Fig. 1:**
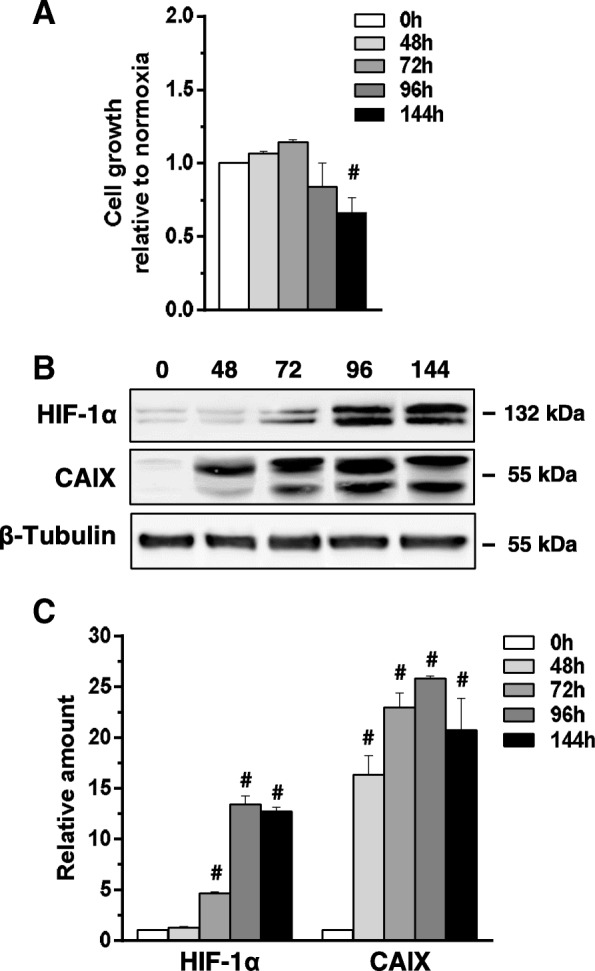
Effects of low oxygen availability on MCF10DCIS cells. **a** Growth of MCF10DCIS cells cultured under low oxygen for the indicated times. The data show the number of viable cells relative to normoxia, taken as 1. **b** Representative Western blot analysis with the indicated antibodies of lysates from MCF10DCIS cells grown at hypoxia for the indicated times. Immunoblots shown have been cropped to conserve space. **c** Levels of HIF-1α and CAIX as deduced from the densitometry of immunochemical bands normalized with β-Tubulin, used as internal control for equivalence of loaded proteins. All the data are the mean of three separate experiments performed in triplicate ±SD. #*P* < 0.05 with respect to normoxia

In order to assess the role of low oxygen availability in breast cancer progression, the effects of hypoxia were investigated in the MCF10DCIS cell line, the only well- established model of DCIS [24]. MCF10DCIS were cultured at normoxia (21% oxygen) or moderate hypoxia (1% oxygen) and evaluated for their malignant properties. As shown in Fig. 1a, low oxygen availability had no effect on cell growth up to 96 h while longer exposure time induced a significant decrease of the number of viable cells. The evaluation of HIF-1α indicated a time dependent accumulation of this master regulator of hypoxia-induced gene transcription (Fig. 1b, c). Because, among the molecules up-regulated by hypoxia through the activity of HIF-1α, the cell surface protein carbonic anhydrase IX (CAIX) is one of the more specific for non-invasive breast cancer cells [25], this protein was evaluated in MCF10DCIS cells cultured under hypoxia. The strong amount of CAIX revealed at all the explored times (Fig. 1b, c) confirmed that our experimental model of low oxygen availability is effective in inducing the hypoxia-related intracellular signalling in MCF10DCIS. On the basis of these preliminary experiments, 96 h have been chosen for evaluation of the effects of low oxygen availability on malignancy of MCF10DCIS.

**Fig. 2:**
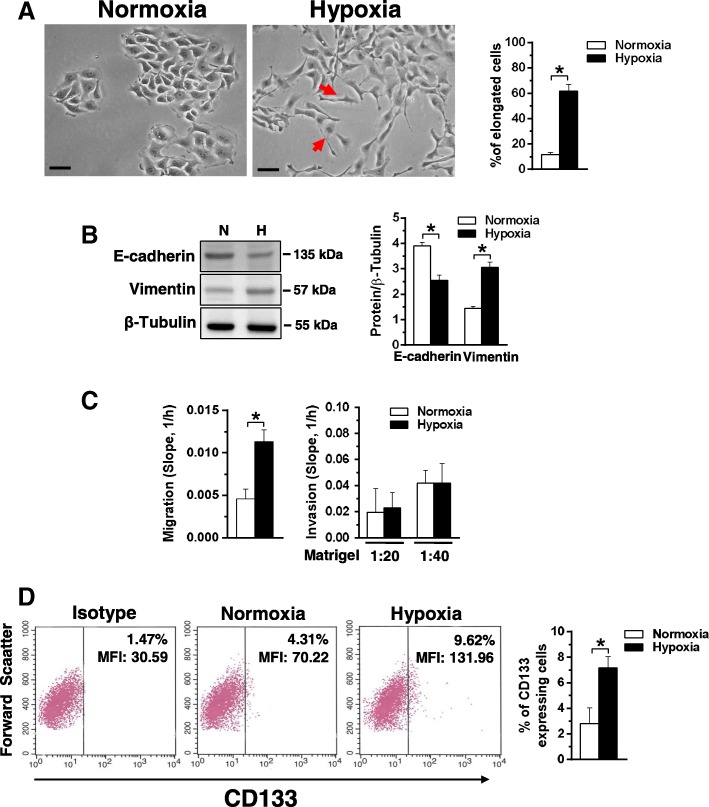
Effects of hypoxia on motility and CD133 expression in MCF10DCIS cells. **a** Representative phase-contrast images of MCF10DCIS cells grown at normoxia or hypoxia for 96 h. The arrows indicate cells with an elongated shape, whose number is shown in the graphs on the right. Bar: 20 μm. **b** Representative immunochemical analysis performed with the indicated antibodies of lysates from MCF10DCIS cells grown at normoxia (N) or hypoxia (H) for 96 h. Immunoblots shown have been cropped to conserve space. On the right, levels of E-cadherin and Vimentin as deduced from the densitometry of immunochemical bands normalized with β-Tubulin, used as internal control for equivalence of loaded proteins. **c** XCELLigence-driven dynamic monitoring of migration and invasion through diluted Matrigel of MCF10DCIS cultured at normoxia or hypoxia for 96 h. The slope analysis, that describes the steepness, incline, gradient, and changing rate of the Cell Index curves over time, was shown. **d** Representative cytofluorimetrical evaluation of CD133 expression with a PE-conjugated antibody in MCF10DCIS cells cultured at normoxia or hypoxia for 96 h. The antigen expression was represented on a bi-parametric dot plot in which a gate was based on the fluorescence emitted after marking with non-specific antibody (Isotype control). The percentage of cells showing high cell surface levels of CD133 is indicated at the upper right of each panel, together with their mean fluorescence intensity (MFI). The mean of three separate experiments ±SD is shown on the right graphs All the data are the mean of three separate experiments performed in triplicate ±SD. *P < 0.05

Phase contrast analysis of cell morphology revealed that 96 h of culture under hypoxic conditions substantially modified the cell shape, with the appearance of cytoplasmic elongations and the acquisition of a spindle-like phenotype (Fig. 2a), suggestive of increased motility.

The ability of low oxygen availability to induce loss of cell-to-cell adhesion, one of the first and crucial stages of malignant progression, was investigated in MCF10DCIS cells cultured under hypoxia by evaluating the canonical EMT markers. As shown in Fig. 2b, MCF10DCIS cells express both proteins and 96 h of culture under low oxygen induced a significant reduction of the epithelial marker E-cadherin and a strong increase of the mesenchymal marker Vimentin.

To assess if the hypoxia-related modifications of morphology and of EMT markers are sufficient to increase migration and/or invasion of MCF10DCIS cells, the Real-Time Cell Analyzer system was used, allowing to demonstrate that 96 h of hypoxia induced a significant increase of the migration capability but had no effect on the invasive properties of this cell line (Fig. 2c).

As it is known that hypoxia may induce the appearance of cells expressing CD133 in breast tumors [23, 26], this cancer stem cell marker was evaluated in MCF10DCIS cultured under low oxygen, showing that the small sub-population of CD133 positive cells detectd at normoxia significantly increased after 96 h of growth under low oxygen availability (Fig. 2d).

### All-*trans* retinoic acid (ATRA) counteracts the effects of hypoxia on DCIS- derived cells

**Fig. 3:**
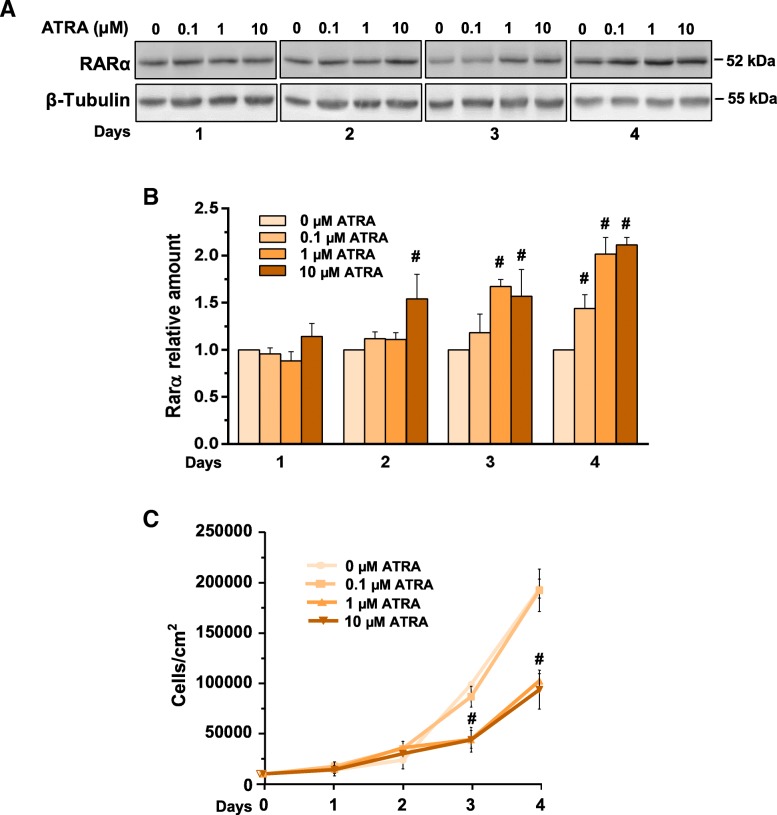
Responsiveness of MCF10DCIS cells to ATRA under normoxia. **a** Representative Western blot analysis with the indicated antibodies of lysates from MCF10DCIS cells grown in the presence of ATRA with indicated concentrations for the indicated times under normoxia. Immunoblots shown have been cropped to conserve space. **b** Levels of RARα as deduced from the densitometry of immunochemical bands normalized with β-Tubulin, used as internal control for equivalence of loaded proteins. **c** Proliferation of MCF10DCIS cells cultured in the presence of different concentrations of ATRA for the indicated times. All the data are the mean of three separate experiments performed in triplicate ±SD. #*P* < 0.05 with respect to the untreated condition taken as 1

At variance with invasive breast cancer derived cells, in which the possible use of ATRA and other retinoids were described to be dependent on tumor phenotypes [27], no data are available on the effects of these drugs in non-invasive breast tumors. Since the rational for the use of retinoids in breast cancer cells requires responsiveness of tumor cells, we firstly established if MCF10DCIS cells are susceptible to ATRA at concentrations commonly used in invasive breast tumor-derived cells. The immunochemical analysis revealed that MCF10DCIS cells express RARα, that significantly increased in a concentration and time related manner as a consequence of ATRA administration (Fig. 3a, b). Following the procedure employed with invasive breast tumor derived cells [27], cell growth was daily monitored, showing that 0.1 μM ATRA was ineffective while 1 μM and 10 μM induced a similar significant decrease of cell proliferation (Fig. 3c), definitely assessing the sensitivity of MCF10DCIS cells to this retinoid.

**Fig. 4:**
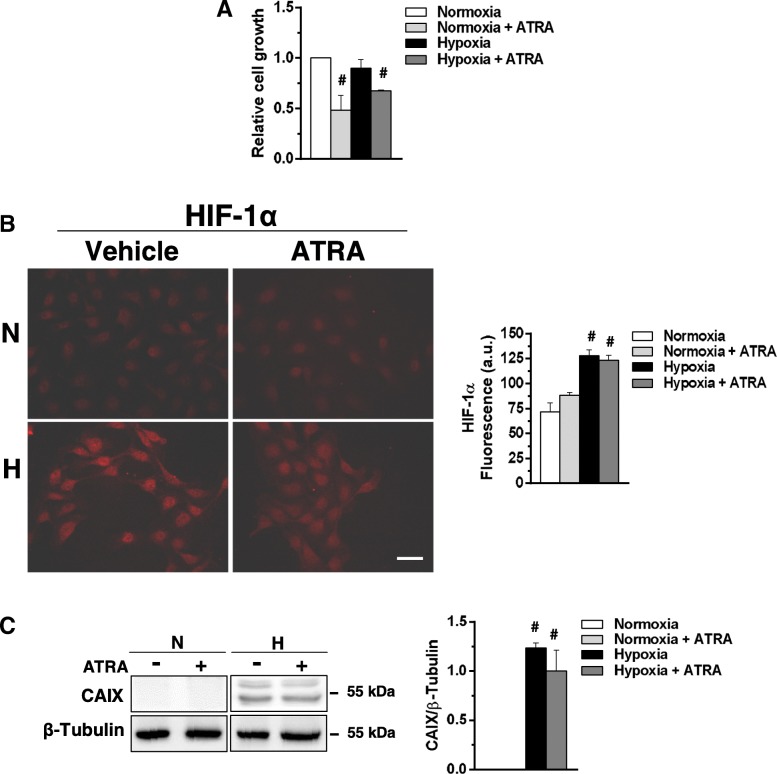
Effects of ATRA on markers of hypoxia in MCF10DCIS cells. **a** Proliferation of MCF10DCIS cells cultured in the presence of 1 μM ATRA for 96 h at normoxia or hypoxia. The data show the number of viable cells relative to normoxia, taken as 1. **b** Representative fluorescence microscopy images of MCF10DCIS cells grown on glass dishes for 96 h at normoxia or hypoxia in the presence of 1 μM ATRA or DMSO (vehicle) and subjected to immunocytochemical analysis with the anti-HIF-1α antibody. Bar: 20 μm. On the right, fluorescence intensity of digitized images calculated by the ImageJ software. **c** Representative Western blot analysis with the indicated antibodies of total lysates from MCF10DCIS cells under the same experimental conditions. Immunoblots shown have been cropped to conserve space. On the right, levels of CAIX as deduced from the densitometry of immunochemical bands normalized with β-Tubulin, used as internal control for equivalence of loaded proteins. Error bars indicate ± SD from a triplicate experiment. **#***P* < 0.05 with respect to normoxia

On the basis of the known role of ATRA in attenuating hypoxia-induced injury in non-trasformed cells [28], its possible role in counteracting the effect of low oxygen availability in non-invasive breast cancer cells was investigated. MCF10DCIS cells were then cultured under moderate hypoxia for 96 h in the presence of 1 μM ATRA, revealing an agonist induced decrease of cell growth similar to that observed under normoxia (Fig. 4a). On the basis of the reported interconnection between HIF-1α and retinoid receptors [29], the effects of ATRA on HIF-1α (Fig. 4b) and on its target CAIX (Fig. 4c) were evaluated, failing to show significant changes under both normoxia and hypoxia.

**Fig. 5:**
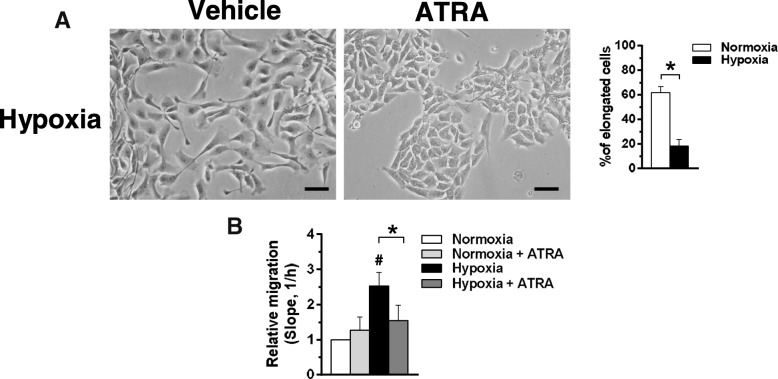
Effects of ATRA on motility of MCF10DCIS cells. **a** Representative phase-contrast images of MCF10DCIS cells grown on plastic dishes under hypoxia for 96 h in the presence of 1 μM ATRA or DMSO (vehicle). The number of cells with an elongated shape is shown on the right. Bar: 20 μm. **b** Dynamic monitoring of migration of MCF10DCIS cultured under normoxic or hypoxic conditions for 96 h in the presence or absence of 1 μM ATRA. Fold changes in the slope compared with normoxia are reported. Error bars indicate ± SD from a quadruplicate experiment. *P < 0.05 between bars; **#***P* < 0.05 with respect to normoxia taken as 1

Concerning cell morphology, cells grown under hypoxia in the presence of ATRA showed a less elongated shape (Fig. 5a), similar to that of cells cultured in normoxia, suggesting that ATRA counteracts the increased motility induced by low oxygen availability in DCIS derived cells. Accordingly, the Real-Time assay of motility of MCF10DCIS cells under the same experimental conditions demonstrated that ATRA significantly reduces their migratory capability (Fig. 5b).

**Fig. 6:**
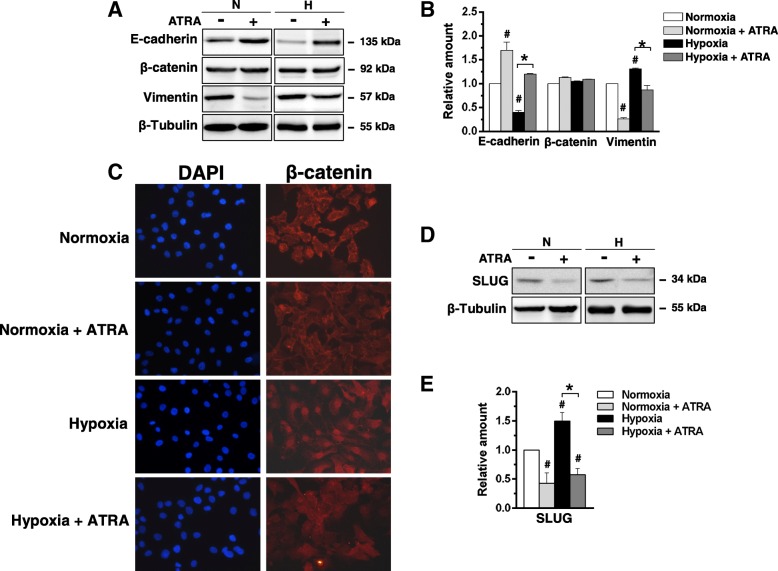
Effects of ATRA on EMT in MCF10DCIS cells. **a** Representative Western blot analysis with the indicated antibodies of lysates from MCF10DCIS cells grown at normoxia (N) or hypoxia (H) for 96 h in the presence or absence of 1 μM ATRA. **b** Relative levels of E-cadherin, β-catenin and Vimentin as deduced from the densitometry of immunochemical bands normalized with β-Tubulin, used as internal control for equivalence of loaded proteins. **c** Representative fluorescence microscopy images of MCF10DCIS cells grown on glass dishes for 96 h at normoxia or hypoxia in the presence or absence of 1 μM ATRA and subjected to immunocytochemical analysis with the anti-β-catenin antibody. Bar: 20 μm. **d** Representative immunochemical analysis performed with the indicated antibodies of lysates from MCF10DCIS cells grown at normoxia (N) or hypoxia (H) for 96 h in the presence or absence of 1 μM ATRA. **e** Relative levels of SLUG as deduced from the densitometry of immunochemical bands normalized with β-Tubulin, used as internal control for equivalence of loaded proteins. Immunoblots shown have been cropped to conserve space. All the data are the mean of three separate experiments performed in triplicate ±SD. *P < 0.05 between bars; **#***P* < 0.05 versus normoxia taken as 1

The immunochemical analysis of the EMT markers revealed that ATRA prevented the effects of hypoxia by inducing a significant increase of E-cadherin and a large decrease of Vimentin (Fig. 6a, b). When a more accurate analysis of the EMT process was performed, we found that, while nor hypoxia neither ATRA affected the expression of β-catenin (Fig. 6a, b), low oxygen availability induced its nuclear accumulation, that was partially inhibited by the administration of the retinoid (Fig. 6c). Accordingly, hypoxia induced the expression of SLUG, that was substantially prevented by ATRA administration (Fig. 6d, e).

**Fig. 7:**
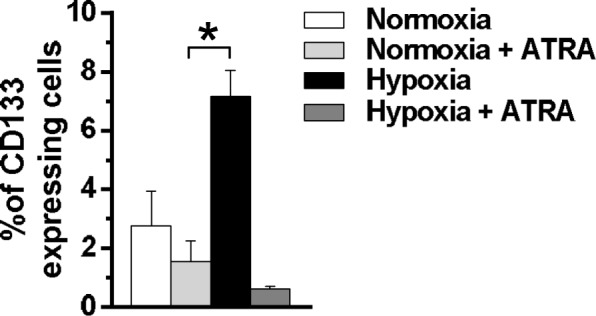
Effects of ATRA on CD133 expression in MCF10DCIS cells. Cytofluorimetrical analysis of CD133 expression in MCF10DCIS cells cultured for 96 h under normoxic and hypoxic conditions in the presence or absence of 1 μM ATRA. A phycoerythrin (PE)-conjugated anti-CD133 antibody was used and surface antigen expression is reported as percentage of positive cells. The data are the mean of three separate experiments performed in triplicate ±SD. *P < 0.05

Since we have above demonstrated that hypoxia induced an increase of MCF10DCIS cells expressing high levels of CD133, this surface antigen was evaluated in cells treated with ATRA during low oxygen exposure. As reported in Fig. 7, ATRA significantly reduced the percentage of CD133 positive cells, almost completely abrogating the up-modulation of this surface antigen induced by hypoxia.

### PLC-β2 mediates the effects of ATRA on CD133 expression in MCF10DCIS cells cultured under hypoxia

**Fig. 8:**
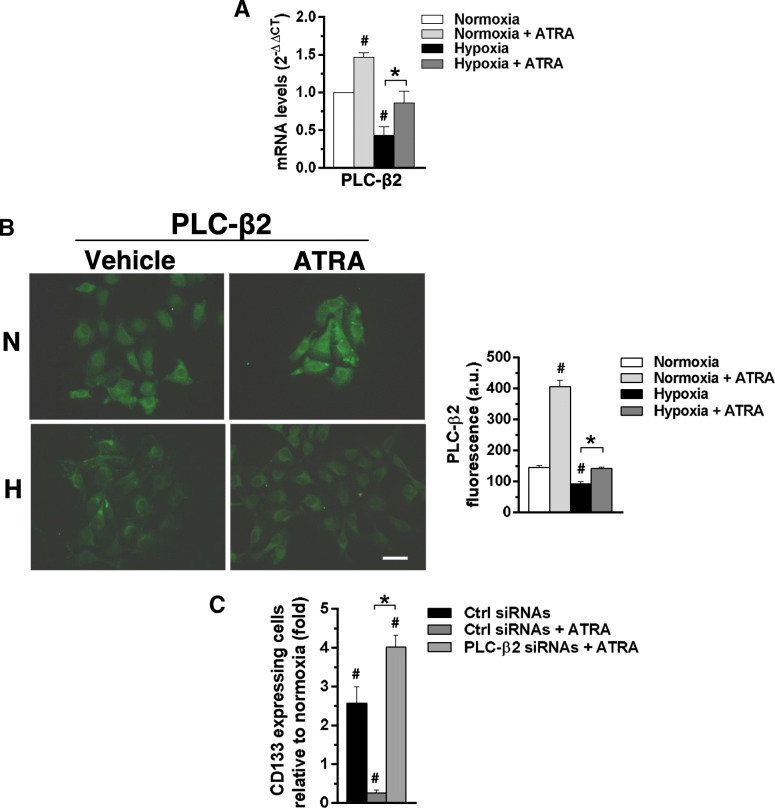
Role of ATRA in modulating PLC-β2 expression in MCF10DCIS cells. **a** Quantitative RT-PCR analysis of PLCβ2 mRNA in MCF10DCIS cells grown for 96 h at normoxia or hypoxia in the presence or absence of 1 μM ATRA. Relative transcript levels were determined using the 2^-△△**Ct**^ method and normalized to RPL13A mRNA. Values represent the fold changes ±SD relative to normoxia, taken as 1. **b** Representative fluorescence microscopy images of MCF10DCIS cells grown on glass dishes for 96 h at normoxia or hypoxia in the presence or absence of 1 μM ATRA and subjected to immunocytochemical analysis with the anti-PLC-β2 antibody. Bar: 20 μm. On the right, fluorescence intensity of digitized images calculated by the ImageJ software. **c** Cytofluorimetrical analysis of CD133 expression in MCF10DCIS cells transfected with siRNAs specific for PLC-β2 (PLC-β2 siRNAs) and cultured for 96 h under hypoxia in the presence or absence of 1 μM ATRA. Scramble siRNAs (Ctrl siRNAs) was used as control. Values represent the fold changes of the percentage of cells expressing high levels of CD133 ± SD relative to normoxia, taken as 1. All the data are the mean of three separate experiments performed in triplicate ±SD. **#***P* < 0.05 versus normoxia; *P < 0.05 between bars

We previously demonstrated that PLC-β2, induced by ATRA in APL-derived cells [30], is down-modulated by low oxygen availability in invasive breast tumor derived cells and that its forced expression reduced the levels of CD133 independently on cell phenotype [21, 23]. Starting from these evidence, a set of experiments was planned to assess the involvement of PLC-β2 in the mechanism by which ATRA counteracts the hypoxia-induced CD133 expression. MCF10DCIS were then cultured for 96 h under low oxygen and both PLC-β2 mRNA and protein amount were evaluated. As shown in Fig. 8a, the mRNA for PLC-β2, quantified by QRT-PCR, significantly decreased after exposure to 1% oxygen. Accordingly, immunocytochemical analysis of cells grown under the same conditions showed a decrease of the protein amount (Fig. 8b). On the other hand, 96 h of 1 μM ATRA induced the expression of PLC-β2 to a significant extent, in terms of mRNA (Fig. 8a) and protein expression (Fig. 8b), in MCF10DCIS cultured in normoxia or under low oxygen.

As determined by flow cytometry, the number of MCF10DCIS cells expressing CD133 at surface level markedly increased as a consequence of the silencing of PLC-β2 during ATRA administration (Fig. 8c), completely abrogating the effects of the retinoid on this surface antigen.

## Discussion

The progression of breast cancer is a very complex and largely unknown phenomenon and seems to depend on various exogenous and endogenous factors [8, 31]. Low oxygen availability, generating a hostile microenvironment in which tumor cells need to activate adaptive mechanisms in order to survive, may have a crucial role in tumor aggressiveness [1, 2, 5]. Accordingly, in breast cancer as in many other solid cancers, low oxygen availability is associated with a clinically aggressive tumor behavior [32].

Even if a non-obligate precursor, DCIS may be a crucial step in progression to invasive ductal carcinoma [31]. Despite hypoxia was reported to promote a cancer-like phenothype in breast epithelial cells [33] and a dedifferentiated phenotype in DCIS [34], no clear correlation between low oxygen availability and malignant progression of non-invasive breast lesions was demonstrated. This study was therefore planned to investigate the role of low oxygenation on malignant properties of MCF10DCIS cells, one of the very few established models of DCIS that, in immunocompromised mice, resulted in rapidly growing lesions that are predominantly high-grade comedo ductal carcinoma in situ [24].

We demonstrated that 96 h of culture at moderate hypoxia are sufficient to induce the epithelial-to-mesenchymal shift, as MCF10DCIS cells cultured under low oxygen loss their epithelial-like shape in favor of a spindle-like phenotype, suggestive of acquired motility. The resulted higher migration capability is in agreement with previous data showing that hypoxia induced the acquisition of migratory and fibroblastoid-like features by polarized non-motile breast derived cells [34]. The nuclear accumulation of β-catenin and the increased expression of its target SLUG, known to have a crucial role in response of breast tumor cells to hypoxia [35], allowed to definitely assess that low oxygen availability induces EMT in DCIS derived cells. Since EMT may be involved in progression from in situ to invasive breast tumors [36], our data confirm that low oxygenation may promote one of the first and most crucial stages of malignant progression in non-invasive breast tumor cells.

The link between hypoxia and cancer stem cells is well documented [37], and cells with a stem-like phenotype were responsible for initiating metastatic growth in various cancers including breast [38, 39]. Increase in stemness induced by hypoxia was reported also in breast tumor cells and tissues [40, 41], and in breast cancer, like in other solid tumors, the expression of the cancer stem cell marker CD133 is associated with low oxygen availability [42]. We revealed here a significant enlargement of the cell population expressing CD133 at surface level in MCF10DCIS cultured under low oxygen, indicating that, in non-invasive as well as in low invasive breast tumor derived cells [21], low oxygen availability induces the appearance of cells with a stem-like phenotype.

Also taking into account the limitation of the study due to the two-dimensional cell culture, this bulk of data, proves the ability of low oxygen availability to promote the EMT process and the appearance of a stem-like phenotype in non-invasive cells, suggesting that drugs acting on hypoxia-related events may prevent malignant progression of non-invasive breast lesions.

In order to counteract the effects of hypoxia in non-invasive breast tumor cells, MCF10DCIS cells were treated with ATRA, a well-known anti-leukemic drug that has been demonstrated to exert anti-tumor roles in cells from invasive breast tumors [15]. In particular, ATRA is reported to induce the formation of adherent junctions and the reorganization of tight junctions by activating a RARα-dependent epithelial differentiation program in sensitive breast tumor cells [16]. ATRA was also reported to promote re-differentiation of early transformed breast epithelial cells [43] and to attenuate hypoxia-induced injury in non-transformed cells [28]. We demonstrated here that the administration of ATRA to MCF10DCIS cells cultured under low oxygen, at concentrations similar to those used with invasive breast tumor-derived cells [27], prevented the mesenchymal shift induced by hypoxia. In agreement with the anti-tumor activity of ATRA in invasive breast tumor, that includes anti-migratory properties [16], we demonstrated that this retinoid hamper the increased motility induced by hypoxia in DCIS derived cells. Finally, in the same cell model, we revealed that ATRA almost completely abrogates the effects of hypoxia on CD133 expression. This suggests that, at least in our in vitro model of non-invasive breast cancer, ATRA is efficient in eliminating hypoxia-induced cells with a stem-like phenotype.

At the basis of the controversial role of ATRA in cancer is the complex retinoid signaling pathway, that regulates the expression of hundreds of genes and modulates a wide variety of fundamental biological processes. In breast cancer, the effects of retinoids seem to depend on which retinoic acid (RA)-inducible genes are expressed as well as on non-genomic effects [17]. In order to elucidate the mechanism by means of which ATRA counteracts the effects of hypoxia in non-invasive breast tumor cell, we have considered the signaling activated by this agonist during differentiation of promyelocytic precursor. We found that PLC-β2, induced by ATRA in APL-derived cells [30], is down-modulated by hypoxia in low invasive breast tumor derived cells [21]. We have also previously demonstrated that PLC-β2 may counteract the expression of CD133 in invasive breast tumor derived cells cultured in normoxia [23] or hypoxia [21], allowing to hypothesize a role for this PLC isozyme in mediating the role of ATRA in DCIS-derived cells cultured under hypoxia. Our data revealed that, as in low invasive breast tumor derived cells, PLC-β2 is ectopically expressed in MCF10DCIS and it is significantly down-modulated as a consequence of low oxygen availability. As in leukemic cells, we found that ATRA induces the expression of PLC-β2 in MCF10DCIS cells, substantially counteracting the decrease of the protein induced by low oxygen exposure. Under hypoxia, we proved that PLC-β2 is essential for the role of ATRA in reducing CD133 expression, demonstrating its crucial role in down-modulating this cancer stem cell marker also in non-invasive breast tumor derived cells. Despite the complexity of the retinoid signaling in breast tumors, our results clearly demonstrate that PLC-β2 is a target for ATRA also in breast tumor cells and is part of the mechanism by means of which this agonist inhibits the acquisition of malignant properties by non-invasive breast tumor-derived cells as a consequence of low oxygen availability. Further results to substantiate the acquired evidence could be obtained by performing the assays on additional non-invasive cell lines with different phenotypes once they will be commercially available.

## Conclusions

Overall, the reported data establish that hypoxia may have a crucial role in the malignant evolution of non-invasive breast lesions and suggest that the administration of ATRA, a well-known anti-tumor drug in leukemia, may be regarded with interest to prevent potential malignant progression of non-invasive breast tumors with hypoxic areas.

## Abbreviations

APL: Acute promyelocytic leukemia
ATRA: All-trans retinoic acid
DCIS: Ductal carcinoma in situ
DMEM F/12: Dulbecco'’s modified Eagle'’s and Ham'’s F-12 media
EMT: Epithelial-to-mesenchymal transition
FBS: Fetal bovine serum
HIFs: Hypoxia-inducible factors
HS: Horse serum
PBS: Phosphate buffered-saline
PE: Phycoerythrin
PLC-β2: Phosphoinositide-dependent phospholipase C β2
RTCA: Real-Time Cell Analyzer
siRNAs: Small interfering RNAs
